# Population based incidence and outcome of surgery for adult patients with hydrocephalus in Sweden

**DOI:** 10.1186/2045-8118-12-S1-O32

**Published:** 2015-09-18

**Authors:** Nina Sundström, Jan Malm, Katarina Laurell, Fredrik Lundin, Babar Kahlon, Kristina Cesarini, Carsten Wikkelsö

**Affiliations:** 1 Department of Radiation Sciences, Biomedical Engineering, Umeå University, Umeå, Sweden; 2 Department of Clinical Neuroscience, Umeå University, Umeå, Sweden; 3 Department of Clinical and Experimental Medicine, Linköping University, Linköping, Sweden; 4 Department of Neurosurgery, University Hospital, Lund, Sweden; 5 Department of Neuroscience, Neurosurgery, Uppsala University, Uppsala, Sweden; 6 Institution of Neuroscience and Physiology, University of Gothenburg, Gothenburg, Sweden

## Background

Incidence and clinical outcome of adult hydrocephalus surgery has never been described in a large population based material. The objective of this study was to present these parameters based on data from the Swedish Hydrocephalus Quality Registry (SHQR). The registry is web-based and contains all adult hydrocephalus patients operated on in four out of five surgical units in Sweden.

## Method

All patients registered in the SHQR during 2004-2011 were included in the study. Data on age, gender, diagnosis and type of surgery were extracted as well as three months outcome for patients with idiopathic normal pressure hydrocephalus (iNPH). Types of surgeries were shunt insertion and endoscopic third ventriculostomy (ETV).

## Results

2360 patients were operated on. Mean total incidence of surgery was 5.1±0.9 surgeries/100000/year; 4.7±0.9 shunt surgeries and 0.4±0.1 ETVs. For iNPH, secondary communicating hydrocephalus and obstructive hydrocephalus the incidence of surgery was 2.2±0.8, 1.9±0.3 and 0.8±0.1 /100000/year respectively. During 2004-2011 the surgical incidence increased in total (p=0.044) and specifically in the age intervals 70-79 years and ≥80 years (p=0.012 and p=0.031). Improvement ≥1 step on the modified Rankin scale (mRS) was seen in 253 of 652 iNPH patients (38.8%) after surgery. Numbers needed to treat was 3.0 for improving one patient from the unfavourable (mRS 3-5) to the favourable (mRS 0-2) group. According to a modified iNPH scale 58% (range 49.3-65.1%, n=704) of the iNPH patients improved. No significant regional differences in incidence, surgical techniques or outcome were found.

## Conclusion

Hydrocephalus surgery increased significantly in frequency in 2004-2011 and specifically in elderly patients. The resulting incidence was however still very low. Surgical treatment for iNPH was a powerful tool for achieving functional independence, as shown by the low NNT, but the clinical outcome was not as good as recently published results. This indicates that outcome reported from clinical studies is not directly comparable to outcome based on everyday hospital assessments among unselected patients representing everyday hospital care.

